# Physicochemical Characterization of Soluble and Insoluble Fibers from Berry Pomaces

**DOI:** 10.3390/gels11100796

**Published:** 2025-10-02

**Authors:** Jolita Jagelavičiūtė, Simona Šimkutė, Aurelija Kairė, Gabrielė Kaminskytė, Loreta Bašinskienė, Dalia Čižeikienė

**Affiliations:** Department of Food Science and Technology, Kaunas University of Technology, Radvilėnų Rd. 19, LT-50254 Kaunas, Lithuania; jolita.jagelaviciute@ktu.lt (J.J.); loreta.basinskiene@ktu.lt (L.B.)

**Keywords:** berry pomace, dietary fiber, soluble fiber, insoluble fiber, technological properties, rheology, viscosity, color parameters, swelling capacity, functional food ingredients

## Abstract

Berry pomace is a valuable source of dietary fiber (DF) with promising applications in functional food development. This study aimed to evaluate and compare the technological and rheological properties of soluble (SDF) and insoluble (IDF) fiber fractions isolated from cranberry, black currant, lingonberry, and sea buckthorn pomace. SDF fractions demonstrated higher water solubility and lower swelling capacity, compared with IDF fractions. Meanwhile, water and oil retention capacities depended on fiber type and the sources of pomace. Fractionation notably affected color parameters, with SDFs generally being lighter. Rheological analysis revealed pseudoplastic, shear-thinning behavior in all SDF samples, with viscosity dependent on both pH and shear rate. In particular, the black currant SDF demonstrated higher yield stress compared to other SDFs, suggesting enhanced resistance to deformation and superior structural stability under low shear conditions. The consistency coefficient varied across samples, indicating differences in gel-forming potential. These findings highlight the importance of berry source and fiber fraction in determining functionality. The distinct hydration, binding, and rheological properties suggest that both SDF and IDF from berry pomace can be strategically applied as thickeners, stabilizers, or texture enhancers in food systems. This study supports the valorization of berry by-products as sustainable and functional ingredients in the formulation of fiber-enriched foods.

## 1. Introduction

Agricultural by-products constitute a significant portion of waste generated within the European Union, amounting to approximately 88 million tons annually [1]. These by-products are increasingly recognized as promising sources of valuable bioactive compounds, including dietary fibers (DF), pigments, essential minerals, fatty acids, and antioxidant polyphenols [2]. Specifically, berry pomace—the residual material obtained following juice extraction—consists primarily of skins, stems, and seeds [1]. The DF fraction in such pomaces is mainly composed of insoluble (IDF) and soluble dietary fibers (SDF), including pectin, lignin, cellulose, hemicellulose, and inulin [1,3].

DFs derived from berry pomace have demonstrated beneficial effects on human health, with evidence supporting their role in reducing the risk of hypertension, obesity, diabetes mellitus, coronary heart disease, stroke, and certain gastrointestinal disorders [1,3]. Current dietary guidelines recommend a total daily fiber intake ranging from 25 to 35 g, with a minimum of 6 g attributable to SDF [1]. Epidemiological studies further indicate that an additional intake of 10 g DF per day may lower the risk of coronary heart disease mortality by up to 35% [1,4].

While IDF contributes primarily to digestive health, its functional and physiological properties differ from those of SDF, which is recognized for its beneficial effects on blood glucose regulation, cholesterol metabolism, gut microbiota modulation, and enhancement of food texture [5]. Despite recommendations by the World Health Organization and the European Food Safety Authority advocating a daily fiber intake of 25 g per individual, actual consumption levels remain suboptimal in many populations [6,7]. Fractionation of DF from berry pomace into SDF and IDF fractions with defined chemical composition and technological properties represents a promising approach to improve the functionality of pomace and facilitate its application in the development of value-added products [8].

Aligned with these health and sustainability objectives, the European Commission launched the Farm to Fork strategy in 2020, which aims to establish a sustainable, equitable, and environmentally sound food system across the European Union. This strategic framework emphasizes the valorization of food industry by-products, promoting their integration as functional ingredients in food products, notably as sources of DF [9,10].

Despite well-documented health benefits and established potential in valorizing berry pomace as a functional food ingredient, significant gaps remain in understanding the technological properties of these by-products. In particular, comparative studies of soluble and insoluble DF fractions from different berry pomaces under standardized conditions are scarce, and comprehensive investigations of their impact on food matrix characteristics and potential applications in novel food formulations are limited. Therefore, this study systematically investigates the yields, composition, and rheological properties and technological properties of SDF and IDF from cranberry, black currant, lingonberry, and sea buckthorn pomace, providing novel comparative data and establishing a scientific basis for their utilization in the development of sustainable, fiber-enriched food products with enhanced nutritional and technological value.

## 2. Results and Discussion

### 2.1. Isolation and Characterization of SDF and IDF from Berry Pomace

The yields of SDF and IDF obtained from cranberry, black currant, lingonberry, and sea buckthorn pomaces are presented in Table 1.

The SDF yield varied considerably among berry types, ranging from 7.4% (sea buckthorn pomace) to 20.71% (cranberry pomace). These differences can be attributed to the distinct composition of berry pomaces, particularly their fiber fractions and associated compounds. Cranberry pomace, which provided the highest SDF yield, has been reported to contain relatively high amounts of soluble fiber [11], whereas sea buckthorn pomace showed lower SDF content and higher proportions of other components. Similarly, lingonberry pomace exhibited the highest IDF yield, while black currant pomace showed the lowest. Variations in SDF and IDF yields are strongly influenced by the intrinsic DF content and the overall chemical composition of the pomace. For instance, Reißner et al. [12] reported that black currant pomace from Germany contained relatively low SDF, while Alba et al. [8] observed considerably higher IDF and SDF contents in black currant pomace from the UK and Poland. Such differences are largely driven by berry variety, climatic and soil conditions, and processing methods, all of which contribute to the observed variation in SDF yield between berry types.

Chemical analysis revealed a relatively high content of proteins and reducing sugars in SDF fractions, suggesting that proteins and low-molecular-weight carbohydrates were not completely removed during the isolation of soluble fiber fractions. In contrast, IDF fractions had significantly lower levels of proteins and reducing sugars. The elevated protein content in the SDF fractions indicates incomplete hydrolysis and insufficient separation of proteins during enzymatic treatment. These findings suggest that, alongside DF, other pomace constituents were also co-extracted, likely due to limitations in the efficiency of the fractionation process. Other studies also reported extracted protein and sugar content in SDF fractions. According to the literature, the yield of DF fractions is strongly influenced by the extraction method [13]. The purity of DF fractions may depend on the fiber source, with some studies reporting purities exceeding 75% after purification [14]. Based on the solubility of DF, purification of SDF using alcohol extraction can be applied. However, the high protein content observed in this study suggests that additional protein hydrolysis may be required. While such procedures can improve fiber purity, they may also considerably increase processing costs, which highlights the importance of evaluating the economic feasibility of scaling up the extraction process when considering industrial applications.

### 2.2. FT-IR of SDF and IDF Fractions

**Figure 1:**
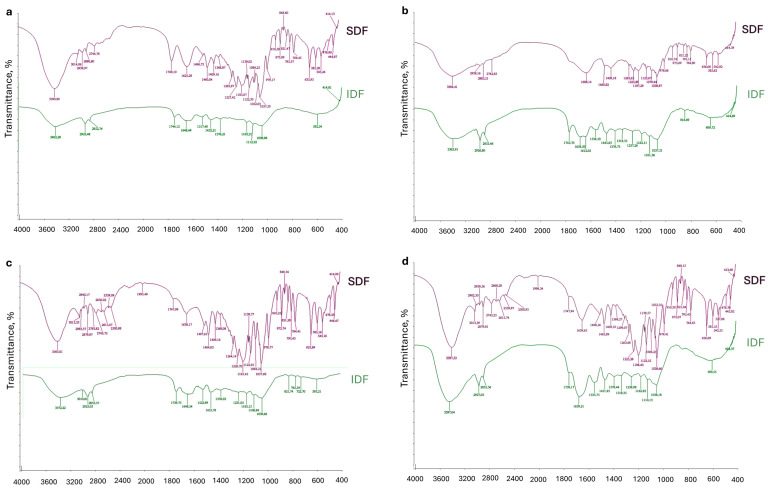
FT-IR spectra of SDF and IDF from cranberry (**a**), black currant (**b**), lingonberry (**c**), and sea buckthorn (**d**) pomaces.

Fourier transform infrared (FT-IR) spectroscopy was employed to identify potential structural differences between the SDF and IDF fractions derived from various berry pomaces. The spectra were recorded within the range of 400–4000 cm^−1^, and the results are presented in Figure 1. Infrared spectroscopy offers valuable insight into the molecular structure of organic compounds, as each exhibits a characteristic “fingerprint” region in its infrared spectrum. This enables the identification of specific compounds through the interpretation of absorption peaks. All analyzed SDF and IDF samples exhibited typical polysaccharide absorption patterns. While the SDF fractions showed similar spectral profiles across different berry sources, variations in peak intensities were observed, reflecting compositional differences. Notably, the SDF fractions presented absorption bands characteristic of pectin within the 1620–800 cm^−1^ region [15,16]. According to Mada et al. [15], the spectral region from 800 to 1200 cm^−1^ corresponds to the fingerprint region of pectins, associated with chemical groups specific to these compounds. Peaks in this range, commonly linked to monosaccharides, reflect vibrations from glycosidic bonds and C–O and C–C linkages within pyranose rings, and thus serve as indicators of structural variation in pectic saccharides [8,17].

Further FT-IR spectral analysis of all SDF and IDF fractions revealed broad absorption bands in the 3200–3600 cm^−1^ range, attributed to O–H and N–H stretching vibrations. These signals indicate the presence of polyhydroxy compounds such as pectin [18], and the extensive hydrogen bonding in this region contributes to the water solubility of DF. The same absorption range may also reflect vibrations typical of cellulose and hemicellulose structures [19,20]. A distinct band around 3011 cm^−1^ was assigned to C–H stretching vibrations [21]. The bands observed between 2830 and 2960 cm^−1^ were mainly attributed to C–H stretching vibrations, and within this interval the −CH, −CH_2_, and −CH_3_ groups of galacturonic acid methyl esters also absorb, confirming the presence of pectic substances [18]. These observations align with findings by Alemdar et al. [22], who reported that the 2800–3600 cm^−1^ region reflects valence vibrations of –CH and –OH groups. Among the detected absorption signals, peaks at approximately 1650 and 1540 cm^−1^ were assigned to proteins [23].

Absorption bands located between 1734 and 1745 cm^−1^ were associated with C=O stretching vibrations of methyl-esterified carboxyl groups. Bands observed between 1612 and 1645 cm^−1^ may correspond to C–O stretching vibrations of conjugated or aromatic ketones typical of lignin. Additionally, peaks in the 1600–1700 cm^−1^ region were attributed to deformation vibrations of carbonyl groups in pectins [19]. A band near 1030 cm^−1^ was attributed to C–OH stretching vibrations characteristic of cellulose and primary alcohols [24].

When comparing different fractions, the sea buckthorn IDF spectrum showed a more pronounced broad absorption band around 3400 cm^−1^, indicating a higher abundance of hydroxyl groups mainly associated with cellulose and hemicellulose. In the black currant SDF spectrum, the O–H stretching band in the 3200–3600 cm^−1^ range appeared somewhat broader than in the other fractions. Cranberry SDF exhibited clearer signals near 1740 cm^−1^ corresponding to carbonyl groups of esterified pectins, whereas lingonberry fractions displayed bands (~1650 and 1540 cm^−1^) attributable to residual proteins. Overall, FT-IR analysis of all SDF and IDF fractions confirmed that the soluble samples are primarily composed of pectin, whereas the insoluble fractions are dominated by lignin and cellulose.

### 2.3. Technological Properties of SDF and IDF Fractions

The technological properties evaluation is essential for DF intended for use in functional foods or as an ingredient in food gels. The SDF and IDF fractions from different berry pomace sources were evaluated, and the results are presented in Table 2. The technological properties of DF varied not only between the SDF and IDF fractions but also depending on the pomace source.

Water retention (WRC) and water swelling capacity (WSC) reflect the fiber’s ability to retain water, a property useful for preventing syneresis and improving the texture of foods [25]. The WRC of SDF ranged from 4.10 to 15.38 g/g d·m., while that of IDF ranged from 4.68 to 10.69 g/g d·m. Among the SDF samples, lingonberry pomace showed the highest WRC, while sea buckthorn pomace exhibited the lowest. The WRC of cranberry, black currant, and lingonberry SDF was higher than that of SDF from apple pomace (3.0–9 g/g) [26], but lower than that of SDF from pummelo and grapefruit cultivars [27]. At the same time, the WRC of IDF of analyzed berry pomace was lower than that of IDF from citrus fruit by-products and pummelo and grapefruit cultivars [27,28]. In the present study, higher residual protein content in SDF fractions was associated with increased WRC. This finding is in line with previous reports showing that protein–pectin associations can enhance water retention in mixed biopolymer networks, as residual proteins provide additional hydrophilic sites and strengthen network interactions [29]. WRC is closely influenced by particle characteristics, which are reflected by bulk density (BD). Higher BD is usually associated with reduced porosity and smaller particle size [30], and in this study, the SDF fractions with the highest BD also demonstrated the greatest WRC.

All SDF fractions exhibited significantly higher water solubility index (WSI) compared to the corresponding IDF fractions. The highest WSI value among SDF fractions was observed in cranberry, while the highest WSI in IDF fractions was found in sea buckthorn. The highest WSI observed in sea buckthorn IDF can be explained by the relatively high content of residual soluble compounds, such as reducing sugars, which contribute to greater solubility despite being part of the insoluble fraction. When evaluating the differences in the technological properties of berry pomace fractions, it is essential to consider the distinct compositional differences between SDF and IDF. The SDF fraction is primarily composed of pectins [31], whose solubility is determined by the presence of free carboxyl groups, forming viscous solutions in water. In contrast, the IDF fraction mainly contains linear (cellulose) and branched (hemicellulose) polysaccharides, as well as lignin, which are poorly soluble in water and generally require high temperatures or specialized treatments for solubilization [25]. Although SDF fractions typically had a higher BD, which is often linked to increased surface area and reduced particle size, the nature of SDF components led to lower WSC and higher WSI. Upon removal of the soluble fraction, the remaining insoluble components demonstrated increased WSC due to the absence of interfering soluble compounds that might otherwise reduce water uptake.

Oil retention capacity (ORC) indicates the fiber’s ability to retain fats or oils, a property that plays a key role in minimizing fat loss and maintaining the stability of high-fat foods and emulsions during processing [25]. The SDF fractions from lingonberry and black currant demonstrated higher ORC compared to their respective IDF fractions. In contrast, cranberry and sea buckthorn pomace showed higher ORC in the IDF fractions than in the SDF. In the present study, SDF fractions with higher residual protein content exhibited elevated ORC, which is consistent with previous findings indicating that proteins contribute hydrophobic sites and interfacial activity that facilitate lipid binding and thereby enhance oil retention [32]. High ORC values are advantageous for minimizing fat loss during food processing and for reducing fat absorption within the intestinal lumen [33]. The enhanced ORC of IDF fractions was attributed to their lower BD. DF particles can adsorb and bind oil components through their non-polar side chains, which interact with hydrocarbon groups of lipids and may also enhance palatability [34]. The ORC values of SDF from black currant and lingonberry were higher than the values previously reported in the literature for SDF from pummelo and grapefruit cultivars [27] and apple pomace [26]. ORC of IDF of cranberry and lingonberry were higher than the reported ORC of IDF from orange (3.62 ± 0.25 g/g), lemon (4.43 ± 0.28 g/g), gonggan (4.42 ± 0.38 g/g), and ponkan (4.41 ± 0.24 g/g) [28]. This comparison highlights the relatively strong oil-binding capacity of berry-derived SDF and IDF. The contrasting patterns observed highlight that ORC is both source- and fraction-dependent, underscoring the importance of compositional characteristics in determining functionality and guiding its potential use in food formulations.

Another important property of DF is color. The color parameters of SDF and IDF fractions from analyzed pomaces differed significantly depending on the fraction and source (Table 3). In most cases, the SDF fraction was brighter compared to the IDF fraction isolated from the same source, except for the SDF from black currant. Among all fractions, the darkest (lowest L* value) were SDF and IDF fractions from black currant pomace, while the lightest were fractions from sea buckthorn.

It can be assumed that a larger content of phenolic compounds remained in the IDF fraction, as the cellulose, hemicelluloses, and lignin present in it form strong complexes with phenolic compounds [35]. Anthocyanins, which are the main red pigments found in black currant press cake, are soluble [36], so a large portion of them could have been washed out by solvents during fractionation. Although the presence of phenolic compounds in IDF fractions may negatively affect color, these compounds also contribute beneficial antioxidant properties [37]. For specific applications where a lighter color is required, additional purification steps (e.g., solvent washing) could be considered, though this may reduce nutritional value. Alternatively, phenolic compounds can be selectively recovered from berry pomace as valuable bioactive co-products, and their separation prior to fiber isolation could both improve the color characteristics of IDF and enhance overall pomace valorization.

### 2.4. Static Rheological Properties of SDF Fraction

**Figure 2:**
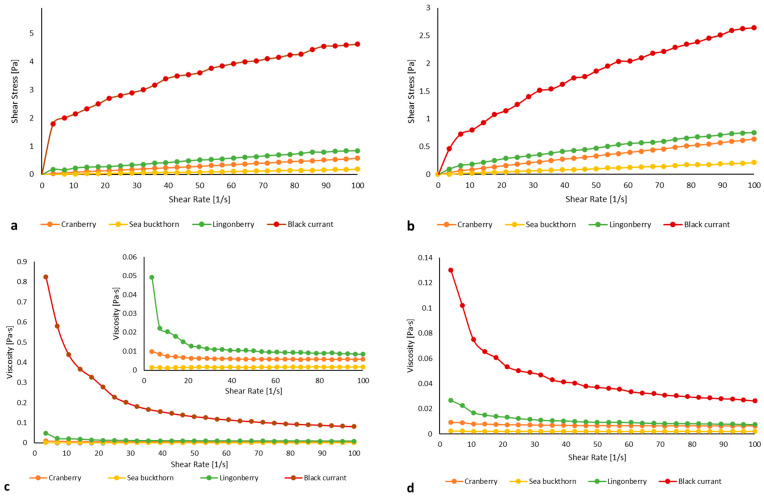
The rheological characteristics of SDF from berry pomace. Shear stress variations under acidic conditions at the inherent pH (cranberry pH 4.7, sea buckthorn pH 4.57, lingonberry pH 4.73, black currant pH 5.14) of each pomace suspension (**a**) and at pH 7.0 (**b**), and viscosity profiles under acidic conditions at the inherent pH of each pomace suspension (**c**) and at pH 7.0 (**d**).

To evaluate the potential of DF for food applications, it is also essential to assess its rheological properties. The variations in shear stress and viscosity as a function of shear rate are presented in Figure 2.

The shear stress increased while the viscosity of all SDF samples decreased progressively with increasing shear rate (Figure 2), indicating typical shear-thinning behavior, characteristic of pseudoplastic non-Newtonian fluids [38]. This trend was confirmed by fitting the data to the Ostwald–de Waele model, with coefficients of determination (R^2^) exceeding 0.90 (Table 4).

In addition, previous studies have shown that high-methoxy pectins, such as those present in apple fiber and citrus pectin, are relatively insensitive to pH reduction or cation presence, but their rheological behavior can be markedly affected by interactions with other food components, such as gelatin [39]. For this reason, all samples were evaluated both at their inherent suspension pH and at a neutral reference (pH 7.0). The suspension pH varied among samples, with black currant SDF showing the highest and sea buckthorn the lowest values (Table 4), reflecting differences in organic acid composition and residual acids retained in the fiber matrix after fractionation [40,41,42]. In the current study, SDFs demonstrated higher shear stress and viscosity under acidic conditions compared to neutral pH (7.0). The molecular interactions between polysaccharide chains are affected by the ionization of hydroxyl and carboxyl groups [43]. Similar trends of decreasing viscosity with increasing pH have also been observed by Yan et al. [44]. FT-IR analysis indicated that the SDF fractions were mainly composed of pectin, which largely determines their rheological behavior. Under alkaline conditions, pectins are susceptible to β-elimination, and the rate of this reaction increases with higher pH values [45]. Consistently, Einhorn-Stoll et al. [46] demonstrated that at pH levels above 6.0, alkaline β-elimination accelerates pectin degradation, resulting in a pronounced decrease in intrinsic viscosity. In line with these observations, the SDF from black currant exhibited the highest shear stress among the samples, which is consistent with its higher viscosity values and may reflect a stronger polysaccharide network supported by extensive hydrogen bonding [42,43,44]. From a nutritional standpoint, increased viscosity has been associated with delayed gastric emptying and a reduced postprandial glucose response, thereby enhancing the functional value of SDF-enriched food formulations [47].

In order to elucidate the rheological characteristics of the SDF fractions, the flow parameters derived from the Ostwald–de Waele model were analyzed. The flow behavior index (*n*) quantifies the degree of non-Newtonian behavior, differentiating between shear-thinning and shear-thickening responses, whereas the consistency index (*K*) is associated with the apparent viscosity and reflects the structural resistance of the system to shear deformation. All samples exhibited a flow behavior index (*n*) below 1 (Table 4), confirming their pseudoplastic nature. Lower *n* values corresponded to more pronounced shear-thinning effects, indicating greater structural adaptability under mechanical stress.

In addition to shear sensitivity, the consistency index (*K*), which reflects the apparent viscosity, varied among the SDFs. The consistency index (*K*) is related to the viscosity characteristics of the fluid. The greater the consistency index, the greater the viscosity. In this study, *K* values ranged from 0.0022 to 1.052 at acidic conditions and from 0.0022 to 0.2389 at pH 7, consistent with the viscosity of liquid or drinkable food products. Such behavior is advantageous in food applications such as sauces, soups, and dressings, where enhanced texture and stability are desirable [48]. The differences in the consistency index (k) among the SDF fractions are likely related to their structural and compositional characteristics. Studies have shown that variations in pectin content, esterification degree, and monosaccharide composition can strongly influence viscosity, leading to higher or lower K values depending on the strength of intermolecular interactions [49,50].

## 3. Conclusions

The comprehensive characterization of dietary fiber fractions obtained from different berry pomaces revealed distinct technological and rheological properties, strongly influenced by both soluble (SDF) and insoluble dietary fiber (IDF) types and the botanical origin of the pomace. SDF fractions exhibited significantly higher water solubility indices and lower water swelling capacity compared to IDF, highlighting their potential use in low-viscosity food applications. In contrast, IDF fractions showed higher swelling and oil retention capacities, suggesting suitability for fat-mimetic or moisture-retaining roles in food matrices.

Color analysis indicated that SDF fractions were generally lighter than IDF. Rheological assessment demonstrated shear-thinning, pseudoplastic behavior of all SDF samples, with flow behavior indices (*n* < 1) and high R^2^ values confirming their non-Newtonian nature. The consistency coefficient (*K*) varied depending on the source, and viscosity was also influenced by pH.

This study provides theoretical guidance for the valorization of berry pomace-derived soluble and insoluble dietary fibers in the development of food systems, particularly gel-based formulations. The technological properties demonstrated—such as water retention, viscosity, and rheological behavior—highlight the potential of SDF fractions as natural structuring agents. Future studies should investigate the role of berry-derived fibers in gel formation dynamics, their molecular interactions within complex food matrices, and their impact on intestinal microbiota, using both in vitro and in vivo models to validate technological functionality and physiological relevance. Their incorporation into food systems also requires evaluation to confirm stability and performance under practical processing conditions and to assess potential in product development. In addition, research on the economic feasibility of large-scale fiber extraction is needed, as this will ultimately determine the practicality of industrial application. Comparative studies with commercially available fibers (e.g., apple, citrus) are recommended to better contextualize the technological value of berry-derived fractions. Finally, given that berry pomace naturally contains both soluble and insoluble dietary fibers, further work should address how recombined SDF and IDF fractions behave in mixed systems to clarify their combined contribution to technological functionality.

## 4. Materials and Methods

### 4.1. Berry Pomaces

Berry pomaces from blackcurrant, lingonberry, cranberry, and sea buckthorn were obtained as local by-products from juice production in Lithuania. All pomaces were processed under standardized conditions to ensure comparable composition and quality for downstream analysis.

All samples were first subjected to supercritical carbon dioxide (SFE-CO_2_) extraction using a Helix system (Applied Separation, Allentown, PA, USA) at 45 MPa, 50 °C, with a CO_2_ flow rate of 2 SL/min for 240 min, in order to remove lipophilic components. These operating conditions were selected based on previously reported optimizations for berry pomace extraction [51]. Following extraction, the material was dried at 35–40 °C for 48 h until the residual moisture dropped below 8%, then ground in a ZM 200 cutting mill (Retsch, Haan, Germany) to a particle size < 500 µm and stored at 4 °C until further analyses.

The proximate composition of berry pomaces used for analysis is summarized in Table 5.

### 4.2. Preparation of IDF and SDF Fractions

The isolation of SDF and IDF fractions was carried out with a modified Total Dietary Fiber assay kit (Megazyme International, Wicklow, Ireland). The procedure followed the manufacturer’s instructions, which are based on AACC Method 32-07.01 and AOAC Method 991.43 [52]. Briefly, pomace sample (50 g) was suspended in 0.05 M MES-Tris buffer (pH 8.2) in a ratio of 1:40 (*w*/*v*) and subjected to enzymatic hydrolysis with thermostable α-amylase (250 µL Megazyme cat. no. E-BLAAM), protease (5000 µL, Megazyme cat. no. E-BSPRT), and amyloglucosidase (10,000 µL, Megazyme cat. no. E-AMGDF) following controlled temperature and agitation conditions. Following enzymatic hydrolysis, the material was centrifuged at 10,000 rpm for 10 min. The pellet was collected and freeze-dried to obtain the IDF fraction. The supernatant was passed through a filter and combined with four volumes of 95% ethanol preheated to 60 °C, then incubated for 1 h to induce SDF precipitation. The resulting suspension was centrifuged again (1000 rpm, 10 min), and the precipitate was rinsed twice with 95% ethanol before being lyophilized to yield the SDF fraction. The recovery of IDF and SDF fractions was determined using Equation (1):

Y (%) = W_1_/W_2_ × 100 (1)

where W_1_ is the mass of the freeze-dried SDF or IDF, and W_2_ is the weight of pomace.

### 4.3. Determination of Chemical Composition

Moisture content was assessed by drying approximately 0.2 g of each sample at 105 °C until constant weight, following AOAC guidelines [53]. Crude protein was measured using the Kjeldahl procedure with a nitrogen-to-protein conversion factor of 6.25, according to AOAC method 978.04 [53]. The concentration of reducing sugars was evaluated using the 3,5-dinitrosalicylic acid colorimetric assay, adapted from the procedure described by Miller [54]. For this, 0.5 g of SDF or IDF was suspended in 100 mL of distilled water, stirred for 10 min, and centrifuged at 1200× *g* for 15 min (Microcen 23, Ortoalresa, Madrid, Spain). An aliquot of 1 mL of the supernatant was combined with 1 mL of DNS reagent and incubated at 95 °C for 5 min. After cooling, 6 mL of distilled water was added, and absorbance was measured at 540 nm using a Genesys 10 spectrophotometer (Thermo Electron LED GmbH, Langenselbold, Germany). The concentration of reducing sugars was calculated using a glucose calibration curve (0–1 mg/mL).

### 4.4. Technological Properties

Hydration properties were evaluated according to Yu et al. [55]. For the determination of water retention capacity (WRC), water swelling capacity (WSC), and water solubility index (WSI), 0.2 g of sample was mixed with 6 mL of distilled water and left to hydrate at room temperature for 18 h. The sample volume was recorded before and after hydration to calculate swelling. Hydrated samples were centrifuged at 1200× *g* for 20 min at room temperature. The residues were weighed, dried at 105 °C to constant mass, and weighed again to determine WRC. The supernatant obtained from centrifugation was dried under the same conditions, and the WSI was calculated.

WRC was calculated based on Equation (2):

WRC (g/g d·m.) = (M1 − M2)/M2(2)

where M1 is the weight of residues before drying (g); M2 is the weight of residues after drying (g).

The water solubility index (WSI) was then determined using Equation (3):

WSI (%) = (M/M0) × 100(3)

where M is the weight of the dried soluble material (g); M0 is the dry pomace weight used prior analysis (g).

WSC was calculated as the increase in volume of the hydrated sample relative to its dry weight, according to Equation (4):

WSC (mL/g d·m.) = (V1 − V0)/M0(4)

where V1 is the volume of the pomace after hydration (mL), V0 is the volume before hydration (mL), and M0 is the dry weight of the pomace prior to hydration (g).

The ORC was determined based on the method of Yu et al. [55], with slight modifications. ORC was determined by mixing 0.2 g of the sample with 2 g of sunflower oil and incubating at room temperature for 1 h. The mixture was centrifuged at 3000 rpm for 10 min, the unretained oil was decanted, and the pellet was weighed. ORC was expressed as

ORC (g/g d·m.) = (W1 − W0)/W0(5)

where W1 is the weight of the pellets (g); W0 is the weight of the sample in dry matter (g).

Bulk density (BD) was determined following Jagelavičiūtė et al. [56]. BD was measured by placing 0.2 g of the sample into a graduated test tube, gently tapping the tube 20 times to compact the sample, and recording the occupied volume. BD was calculated using Equation (6).

BD (g/mL d·m.) = M/V(6)

where M is the weight of the sample in dry matter (g); V is the sample volume occupied in the test tube (mL).

Color parameters were assessed with a Konica Minolta colorimeter (Japan) using the CIE Lab system. In this scale, L* represents brightness from 0 (black) to 100 (white), a* indicates the position on the green–red axis, and b* corresponds to the blue–yellow axis. For analysis, the sample was evenly spread across a plate, and measurements were taken randomly from three different positions at the bottom of the plate.

### 4.5. FT-IR

FT-IR spectra of SDF and IDF fractions were collected using a Perkin Elmer Spectrum GX 2000 spectrometer (Perkin–Elmer Ltd., Beaconsfield, Buckshire, UK) equipped with a single-reflectance horizontal ATR cell containing a diamond crystal. The samples were mixed with KBr in a 1:100 (*w*/*w*) ratio and analyzed in transmission mode over the range of 4000–400 cm^−1^.

### 4.6. Determination of Static Rheological Properties

Static rheological properties of SDF were evaluated according to Chen et al. [43]. Measurements were carried out on a Physica MCR101 rheometer (Anton Paar GmbH, Graz, Austria) fitted with a concentric cylinder system. The measuring geometry included a cup (27 mm inner diameter, 35 mm outer diameter) and a bob with a recessed bottom (24.003 mm diameter, 24.938 mm height).

To assess the effect of pH, sample dispersions (10 g/100 mL) were prepared using distilled water. Two variants were tested: (i) without pH adjustment, and (ii) with pH adjusted to 7.0 using 1 M NaOH. All dispersions were left to hydrate under continuous stirring for 1 h at room temperature before measurements.

Flow curves were obtained by measuring shear stress (τ) as a function of shear rate ($\dot{\gamma }$) in the range of 0–100 s^−1^. The data were analyzed using the Ostwald–de Waele (power-law) model (Equation (7)), and the fitting precision was expressed as R^2^:

(7) $$\tau \;=\;K\cdot (\dot{\gamma }{)}^{n}$$

where τ is the shear stress (Pa), $\dot{\gamma }$ is the shear rate (s^−1^), K is the consistency index (Pa·s^n^), *n* is the flow behavior index (dimensionless).

Apparent viscosity (η_50_) at a shear rate of 50 s^−1^ was calculated from the model equation.

### 4.7. Statistical Analyses

All measurements were performed in triplicate, and results are reported as mean ± standard deviation, calculated in Microsoft Excel 2019 (Microsoft Corp., Albuquerque, NM, USA). Statistical evaluation was carried out using Statgraphics Centurion 19. Differences among groups were tested by one-way ANOVA, followed by Tukey’s HSD post hoc test, with significance accepted at *p* ≤ 0.05.

## Figures and Tables

**Table 1:** The yield and compositions of SDF and IDF extracted from different berry pomaces.

DF Source		Yield, %	Moisture, %	Protein, g/100 g d·m.	Reducing Sugars, g/100 g d·m.
Cranberry	SDF	20.71 ± 0.91 c	8.19 ± 0.22 c	24.17 ± 0.2 f	9.15 ± 0.2 f
	IDF	66.12 ± 2.94 e	4.55 ± 0.21 a	3.92 ± 0.03 b	2.0 ± 0.03 a
Black currant	SDF	13.12 ± 0.60 b	9.42 ± 0.57 d	29.09 ± 0.2 g	12.57 ± 0.4 g
	IDF	46.96 ± 1.82 d	5.71 ± 0.76 b	8.67 ± 0.5 e	6.58 ± 0.1 c
Lingonberry	SDF	15.80 ± 0.64 b	8.42 ± 0.26 cd	39.67 ± 0.2 h	8.52 ± 0.2 e
	IDF	70.60 ± 2.36 e	4.71 ± 0.23 ab	4.08 ± 0.4 c	3.81 ± 0.04 b
Sea buckthorn	SDF	7.40 ± 0.29 a	9.12 ± 0.32 cd	5.62 ± 0.03 d	11.91 ± 0.3 g
	IDF	68.00 ± 2.45 e	5.21 ± 0.21 b	2.84 ± 0.02 a	7.97 ± 0.1 d

Mean ± standard deviation values in column with different lowercase letters are significantly different (*p* < 0.05).

**Table 2:** Technological properties of SDF and IDF fractions from different berry pomaces.

Dietary Fiber Source		WRC, g/g d·m.	WSC, mL/g d·m.	ORC, g/g d·m.	WSI, %	BD, g/mL d·m.
Cranberry	SDF	8.95 ± 0.10 d	1.10 ± 0.0 b	2.64 ± 0.12 b	71.8 ± 0.6 g	0.32 ± 0.01 c
	IDF	10.69 ± 0.36 e	2.52 ± 0.06 e	7.24 ± 0.32 f	4.8 ± 0.3 a	0.13 ± 0.01 a
Black currant	SDF	13.04 ± 0.1 f	2.20 ± 0.0 d	5.48 ± 0.2 d	64.1 ± 0.1 e	0.32 ±0.0 c
	IDF	9.60 ± 0.6 de	3.15 ± 0.0 f	3.34 ± 0.2 c	4.5 ± 0.1 a	0.17 ± 0.0 b
Lingonberry	SDF	15.36 ± 0.08 g	0.55 ± 0.0 a	15.36 ± 0.08 g	69.42 ± 0.2 f	0.36 ± 0.0 d
	IDF	6.53 ± 0.03 c	1.57 ± 0.01 c	6.53 ± 0.03 e	7.85 ± 0.0 b	0.19 ± 0.0 b
Sea buckthorn	SDF	4.10 ± 0.05 a	2.81 ± 0.21 e	1.62 ± 0.03 a	20.7 ± 0.1 d	0.31 ± 0.1 c
	IDF	4.68 ± 0.21 b	3.82 ± 0.16 g	1.70 ± 0.07 a	12.1 ± 0.3 c	0.34 ± 0.1 cd

Mean ± standard deviation values in columns with different lowercase letters are significantly different (*p* < 0.05).

**Table 3:** Color parameters of SDF and IDF fractions from different berry pomaces.

Dietary Fiber Source		L*	a*	b*
Cranberry	SDF	58.63 ± 0.41 f	12.78 ± 0.07 g	15.20 ± 0.04 e
	IDF	46.37 ± 0.21 d	14.28 ± 0.05 h	11.68 ± 0.02 c
Black currant	SDF	23.64 ± 0.20 a	8.14 ± 0.08 e	2.80 ± 0.05 a
	IDF	37.36 ± 0.02 b	8.62 ± 0.01 f	3.93 ± 0.01 b
Lingonberry	SDF	56.36 ± 0.22 e	0.91 ± 0.0 a	19.12 ± 0.04 g
	IDF	37.61 ± 0.13 c	1.22 ± 0.0 b	18.71 ± 0.02 f
Sea buckthorn	SDF	65.64 ± 0.12 g	5.59 ± 0.01 d	12.76 ± 0.04 d
	IDF	56.66 ± 0.02 e	5.20 ± 0.00 c	21.85 ± 0.01 h

Mean ± standard deviation values in column with different lowercase letters are significantly different (*p* < 0.05).

**Table 4:** Ostwald–de Waele model parameters for SDF aqueous suspensions.

DF Source	pH	*K* (Pa s*^n^*)	*n*	*η*_ap_ (Pa s)	R^2^
Cranberry	4.70 ± 0.01 b	0.0099 ± 0.0004 c	0.528 ± 0.02 b	0.0058 ± 0.0004 c	0.9990
	7.00 ± 0.01 d	0.0053 ± 0.0002 b	0.718 ± 0.01 e	0.0063 ± 0.0001 d	0.9990
Black currant	5.14 ± 0.02 c	1.0520 ± 0.083 g	0.317 ± 0.015 a	0.0729 ± 0.0090 g	0.9810
	7.00 ± 0.03 d	0.2389 ± 0.0052 f	0.524 ± 0.005 b	0.0371 ± 0.0003 f	0.9970
Lingonberry	4.73 ± 0.01 b	0.0579 ± 0.0047 e	0.563 ± 0.018 c	0.0103 ± 0.0007 e	0.9463
	7.00 ± 0.02 d	0.0428 ± 0.0023 d	0.621 ± 0.007 d	0.0097 ± 0.0005 e	0.9970
Sea buckthorn	4.57 ± 0.02 a	0.0022 ± 0.0001 a	0.925 ± 0.034 f	0.0016 ± 0.0001 a	0.9920
	7.00 ± 0.03 d	0.0022 ± 0.00001 a	0.992 ± 0.009 g	0.0021 ± 0.0002 b	0.9980

*K*, consistency index (Pa s*^n^*); *n*, flow behavior index; *η*_ap_, shear viscosity at 50 s^−1^ shear stress. Mean ± standard deviation values in column with different lowercase letters are significantly different (*p* < 0.05).

**Table 5:** Proximate composition of berry pomaces used for analysis.

Pomace	SDF, g/100 d·m.	IDF, g/100 g d·m.	Proteins, g/100 g d·m.	Lipids, g/100 g d·m.	Ash, g/100 g d·m.
Cranberry	12.24 ± 0.54	61.78 ± 0.22	7.60 ± 0.09	7.13 ± 0.39	1.00 ± 0.01
Black currant	8.85 ± 0.35	42.20± 0.90	9.74 ± 0.46	8.67 ± 0.10	4.02 ± 0.03
Lingonberry	8.94 ± 0.15	68.26± 1.47	9.03 ± 0.01	7.60 ± 0.04	1.24 ± 0.01
Sea buckthorn	6.72 ± 0.44	63.93± 0.68	25.45 ± 0.41	1.80 ± 0.20	1.53 ± 0.03

## Data Availability

The original contributions presented in this study are included in the article. Further inquiries can be directed to the corresponding author.

## Abbreviations

The following abbreviations are used in this manuscript:

BD	Bulk density
DF	Dietary fiber
FT-IR	Fourier transform infrared
IDF	Insoluble dietary fiber
ORC	Oil retention capacity
K	Consistency index
SDF	Soluble dietary fiber
WRC	Water retention capacity
WSC	Water swelling capacity
WSI	Solubility index
*n*	flow behavior index
*η*_ap_	apparent viscosity
